# Multifunctional Bamboo Fiber/Epoxy Composites Featuring Integrated Superhydrophobicity and Enhanced Mechanical–Thermal Performance

**DOI:** 10.3390/nano16010008

**Published:** 2025-12-19

**Authors:** Yanchao Liu, Ze Yu, Rumin Li, Xiaodong Wang, Yingjie Qiao

**Affiliations:** College of Material Science and Chemical Engineering, Harbin Engineering University, Harbin 150001, China

**Keywords:** bamboo, epoxy resin, alkali treatment, hydrophobic coating

## Abstract

Developing sustainable, high-performance biomass composites is crucial for replacing non-renewable structural materials. In this study, a “bamboo steel” composite was fabricated using a multilevel modification strategy involving alkali pretreatment, toughened resin impregnation, and surface functionalization. Bamboo fibers were treated to remove hemicellulose and lignin, enhancing porosity and interfacial bonding. The bamboo scaffold was subsequently impregnated with a thermo-plastic polyurethane-modified epoxy resin to create a robust, interpenetrating network. The optimized composite (treated at 80 °C) exhibited a flexural strength of 443.97 MPa and a tensile strength of 324.14 MPa, demonstrating exceptional stiffness and toughness. Furthermore, a superhydrophobic coating incorporating silica nanoparticles was applied, achieving a water contact angle exceeding 150° and excellent self-cleaning properties. This work presents a scalable strategy for producing bio-based structural materials that balance mechanical strength with environmental durability.

## 1. Introduction

With the advancement of global sustainable development strategies and the implementation of “dual carbon” targets, the development of green, lightweight, and high-strength bio-based composites has become a key research focus, aiming to replace non-renewable conventional materials such as metals and plastics. Traditional structural materials—including metals, ceramics, concrete, and polymer composites—are generally non-renewable, often have high density, and may cause adverse environmental impacts [1,2]. Accordingly, there is growing urgency to develop renewable and eco-friendly biomaterials that can replace synthetic counterparts in applications ranging from civil engineering to the aerospace industry. In response to environmental concerns associated with synthetic fiber composites, researchers have increasingly turned their attention to the potential of natural biomass fibers [3].

Bamboo is a highly promising biomass material owing to its rapid growth, renewability, and exceptional mechanical properties—such as high specific strength and a distinctive microstructure [4,5,6,7]. As a green reinforcement, bamboo offers an excellent balance of strength, flexibility, and low environmental impact [8]. Its natural esthetics, combined with low-carbon and eco-friendly characteristics, further enhance its appeal, positioning it as a sustainable alternative for a variety of applications [9]. Compared with most commercial wood species, bamboo grows faster and possesses a unique structure composed of vascular bundles embedded in a matrix of thin-walled cells [10]. These vascular bundles are rich in bamboo fibers, endowing the material with physical and mechanical properties comparable or even superior to those of wood. This makes bamboo an increasingly attractive alternative in the context of growing timber scarcity [11,12]. However, natural bamboo suffers from inherent limitations, including pronounced anisotropy, high hygroscopicity, poor dimensional stability, and interfacial incompatibility caused by its abundant hydroxyl groups and porous structure. These drawbacks restrict its application in high-performance structural materials [13]. In recent years, ionic liquids have gained attention as an effective medium for dissolving cellulose, while simultaneously facilitating the curing of epoxy resins. This approach offers an alternative method for enhancing fiber-matrix interfacial bonding and improving the mechanical properties of cellulose-based composites. While these ionic liquid-based methods show promise, they often involve complex processing steps and additional environmental considerations [14,15].

“Impregnation reinforcement” has been proposed as an effective modification strategy [16]. Epoxy resin (EP), known for its excellent adhesion, mechanical properties, and chemical resistance, is widely used as an impregnating matrix [17]. However, although cured epoxy resins possess high strength, they are inherently brittle and susceptible to cracking under impact. This brittleness can induce stress concentration at the bamboo–resin interface, leading to insufficient composite toughness. Thermoplastic polyurethane (TPU), an elastomer with a microphase-separated structure that combines the strength of plastics with the elasticity of rubber, has been widely employed as a toughening modifier for epoxy resins [18,19,20,21]. Incorporating TPU into EP for bamboo impregnation is expected to preserve high strength while significantly improving toughness, thereby achieving a better balance between rigidity and ductility [22,23,24].

Moreover, pretreatment of bamboo substrates plays a critical role in optimizing the impregnation process. Alkali treatment, such as with NaOH solution, effectively removes partial lignin and hemicellulose, increases porosity, and reduces hydroxyl content, thereby lowering hydrophilicity and creating more favorable interfacial sites and space for polymer impregnation [25,26]. While existing studies have acknowledged that alkali treatment regulates the multi-scale porosity of bamboo and exposes active functional groups-affecting both resin infiltration and interfacial stress transfer-the extent and mechanism of these effects remain insufficiently investigated. Building on this, constructing a hydrophobic coating using 1H,1H,2H,2H-heptadecafluorodecyltrimethoxysilane (FAS-17) and fumed silica can further enhance composite performance. The low surface energy of FAS-17 reduces surface tension and imparts hydrophobicity, while fumed silica increases surface roughness, synergistically enhancing the hydrophobic effect [27,28,29]. Although surface treatments have been applied individually in previous studies, their integration with simultaneous structural tuning and interfacial toughening within a single composite system has rarely been reported.

**Figure 1:**
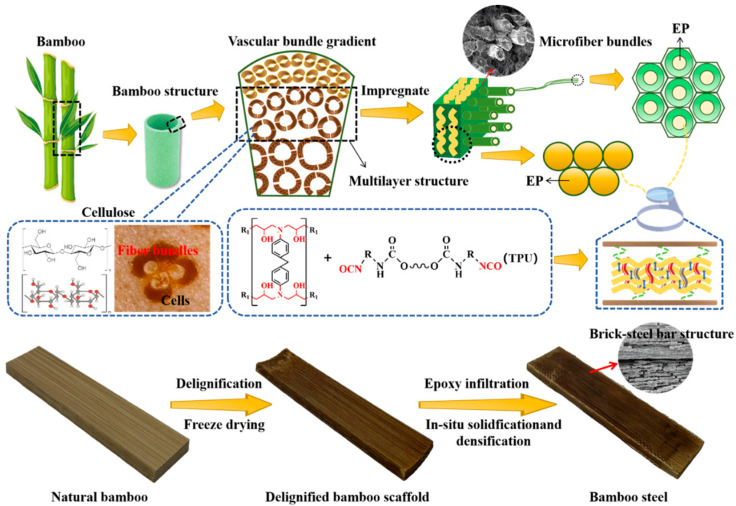
Flowchart of preparation composites.

In this study, natural bamboo was subjected to delignification and partial hemicellulose removal to obtain porous bamboo fibers, which were subsequently aligned and impregnated with thermoplastic polyurethane (TPU)-modified epoxy resin (EP-TPU). The alkali-treated bamboo–epoxy composites were fabricated via hot-press molding, as schematically illustrated in Figure 1 (the detailed preparation procedure is provided in the Supporting Information). During hot pressing, the EP-TPU matrix sufficiently infiltrated the hierarchical pores of the bamboo fibers, forming an interpenetrating “reinforced concrete-like” network that facilitates efficient stress transfer from the matrix to the fibers. Furthermore, a superhydrophobic coating was applied by spraying a mixture of FAS-17 and fumed silica onto the composite surface, imparting excellent water resistance and self-cleaning performance. By simultaneously tailoring the internal bamboo scaffold, the fiber–matrix interface, and the external surface, this work establishes a multilevel modification framework that enhances stiffness, toughness, and environmental durability in a coordinated manner. This integrated approach provides a more comprehensive reinforcement strategy compared with conventional single-level modification methods for bamboo composites. Owing to the abundance and renewability of bamboo, these reinforced concrete-like composites, which exhibit superior mechanical performance, show significant potential as sustainable structural materials for applications in civil engineering, automotive interiors, wind turbine blades, and aerospace.

## 2. Materials and Methods

Prior to composite fabrication, alkali-treated bamboo (ABF) was prepared to enhance interfacial compatibility. Natural bamboo was immersed in a 0.2 mol/L NaOH solution at varying temperatures (40, 60, 80, and 100 °C) for 12 h to partially remove lignin and hemicellulose. The treated bamboo was subsequently rinsed with deionized water until neutral and dried in a vacuum oven at 40 °C for 12 h. For the resin matrix, Thermoplastic polyurethane elastomer (TPU, Shanghai Aladdin Biochemical Technology Co., Ltd., Shanghai, China, 15 g) was dissolved in *N*,*N*-Dimethylformamide (DMF, Shanghai Aladdin Biochemical Technology Co., Ltd., 25 g) at 120 °C with stirring until complete dissolution. Subsequently, epoxy resin (E-51, Sinopec, Beijing, China, 100 g) was added, and stirring was continued for 3 h to achieve thorough mixing. During this process, the system changed from colorless transparent to orange-red transparent. After cooling to room temperature, the TPU-modified epoxy resin system was obtained and set aside for later use. 100 g of the above TPU-modified epoxy resin system was mixed with 4,4’-diaminodiphenylmethane (DDM, Shanghai Aladdin Biochemical Technology Co., Ltd., 25 g) and stirred at 60 °C until DDM was fully dissolved. The alkali-treated bamboo was immersed in the resulting mixture and subjected to vacuum impregnation for 12 h. Afterwards, hot-pressing was carried out at 120 °C under 10 MPa to form the final alkali-treated bamboo–epoxy resin composite. Detailed procedures are provided in the Supporting Information.

## 3. Results and Discussion

### 3.1. Structural Characterization

**Figure 2:**
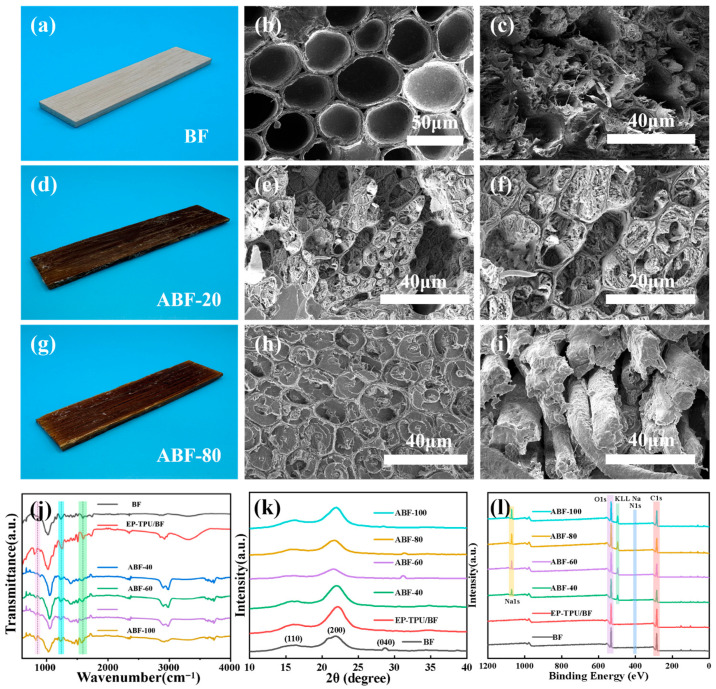
(**a**,**d**,**g**) Photographs, (**b**,**e**,**h**) Cross-sectional SEM images and (**c**,**f**,**i**) Fracture surface SEM images of BF, EP-TPU/BF and ABF-80; (**j**) FTIR spectra, (**k**) XRD patterns and (**l**) XPS survey spectra of BF, EP-TPU/BF, ABF-40, ABF-60, ABF-80 and ABF-100.

The color changes observed after impregnation (Figure 2a–c) reflect the incorporation of the TPU-modified epoxy into the bamboo substrate. The untreated BF sample (Figure 2d) exhibits slight deformation due to bending but no fiber fracture or pull-out, indicating limited intrinsic toughness. In contrast, ABF-20 (Figure 2e) displays thick cell walls, distinct voids, and interfacial gaps, confirming that the dense lignin–hemicellulose network restricts resin penetration. Such incomplete impregnation compromises stress transfer and interfacial integrity. A markedly different morphology is obtained for ABF-80 (Figure 2f,i). Alkali treatment partially removes lignin and hemicellulose, producing thinner cell walls and additional microchannels [30]. These modifications enhance resin accessibility, resulting in fully filled lumens and minimal voids. The improved wetting and mechanical interlocking are consistent with a more efficient stress-transfer pathway and better composite performance.

FT-IR spectra (Figure 2j) corroborate these structural changes. The lignin-related bands at 1506 and 1593 cm^−1^ progressively decrease with increasing alkali-treatment temperature, indicating gradual lignin removal [31,32]. The band at 1235 cm^−1^, associated with hemicellulose and lignin, weakens and disappears under stronger alkaline conditions [33]. EP-TPU/BF exhibits characteristic polyurethane features, including N–H stretching (3320 cm^−1^), ester C=O stretching (1730 cm^−1^) [34], the amide II band (1660 cm^−1^), and C–O–C and C–N absorptions at 1140 and 1050 cm^−1^ [35]. XRD analysis (Figure 2k) shows typical cellulose I reflections for BF. A slight shift to higher 2θ in EP-TPU/BF suggests a denser packing of the cellulose chains, likely due to the compressive forces exerted by the curing resin matrix, resulting in reduced interplanar spacing. Alkali-treated samples display gradual shifts toward lower angles with increasing treatment severity. ABF-100 exhibits the largest shift and peak broadening, indicative of partial crystalline disruption. ABF-80 shows a moderate shift while retaining a relatively sharp peak, suggesting removal of amorphous components (primarily hemicellulose and lignin, which lack long-range order) with preservation of the crystalline cellulose framework. This balanced modification improves surface roughness and wetting, supporting enhanced interfacial bonding [36]. XPS results (Figure 2l) further support the chemical evolution. Pristine BF contains only C 1s and O 1s signals. After impregnation, EP-TPU/BF displays a clear N 1s peak, confirming the introduction of TPU-derived urethane groups. With increasing alkali-treatment temperature, Na-related signals (Na KLL and Na 1s) become more pronounced, consistent with greater exposure of oxygen-containing functional groups capable of binding Na^+^ [37]. At higher temperatures (80–100 °C), stronger Na signals imply deeper surface modification. The increase in the O/C ratio and the emergence of Na Auger signals indicate the exposure of active hydroxyl groups and the formation of sodium cellulosate intermediates. This enhanced surface polarity facilitates better wetting by the polar epoxy matrix [38].

### 3.2. Mechanical Properties of Alkali-Treated Bamboo Composites

**Figure 3:**
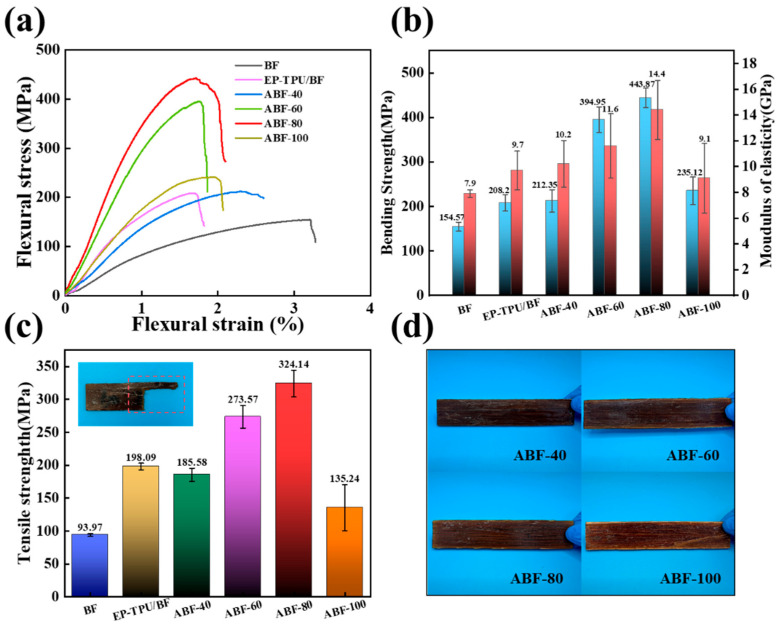
(**a**) Bending stress–strain curves, (**b**) Bending strength and elastic modulus, (**c**) Tensile strength and (**d**) Photographs of BF, EP-TPU/BF, ABF-40, ABF-60, ABF-80 and ABF-100.

Figure 3 and Table 1 summarize the mechanical behavior of BF and alkali-treated composites. The bending stress–strain curves (Figure 3a) and the corresponding flexural strength and modulus (Figure 3b) show a consistent trend: both properties increase with alkali-treatment temperature up to 80 °C, followed by a decrease at 100 °C. The ABF-40 sample exhibits only a slight improvement relative to EP-TPU/BF, indicating limited enhancement of the fiber–matrix interface. Treatments at 60 °C and 80 °C lead to more substantial gains, consistent with the more effective removal of hemicellulose and lignin at these temperatures, which increases surface roughness and improves resin wetting and interfacial bonding [39]. The ABF-80 composite shows the highest flexural performance, achieving a flexural strength of 443.97 MPa and a modulus of 14.4 GPa-corresponding to increases of 213% and 148%, respectively, compared with EP-TPU/BF. At 100 °C, however, over-etching of the fiber cell walls results in structural degradation and reduced interfacial integrity, which explains the decline in mechanical properties. A similar trend is observed for tensile behavior (Figure 3c). The tensile strength of ABF-80 reaches 324.14 MPa, representing a 163.6% improvement over EP-TPU/BF, further supporting the conclusion that interfacial bonding dominates the load-transfer efficiency in these composites. The macroscopic appearance of the composites (Figure 3d) is consistent with the mechanical data. The ABF-60 and ABF-80 specimens show smooth surfaces and uniform resin distribution, while the ABF-100 sample displays visible fiber exposure and interfacial delamination, indicative of excessive alkaline degradation. These results demonstrate that alkali treatment at 80 °C provides the most effective balance between component removal and structural preservation, yielding optimal mechanical performance in TPU-modified bamboo fiber composites.

### 3.3. Dynamic Mechanical Analysis (DMA)

**Figure 4:**
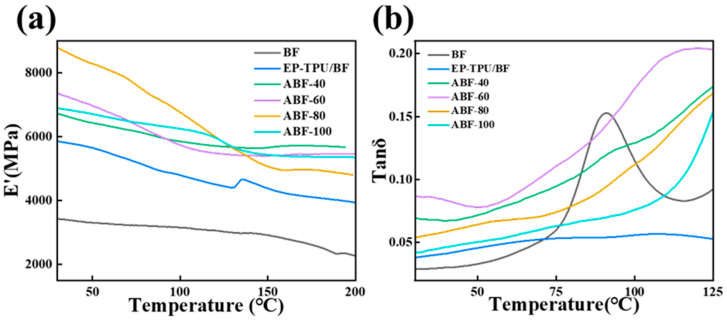
(**a**) Storage modulus (E′) as a function of temperature and (**b**) tanδ of BF, EP-TPU/BF, ABF-40, ABF-60, ABF-80, and ABF-100.

DMA was conducted to examine the influence of alkali-treatment temperature on the thermo-mechanical behavior of EP-TPU/BF composites. As shown in Figure 4a, all samples exhibit a gradual reduction in storage modulus (E′) with increasing temperature due to matrix softening. Differences among the composites are small at temperatures below ~40 °C, indicating that mild alkaline treatment (40–60 °C) causes only limited modification to the fiber surface. More noticeable separation between curves appears from 40 to 120 °C, where the ABF-60 and ABF-80 composites show slightly higher E′ values than untreated or over-treated counterparts. This trend suggests that moderate lignin/hemicellulose removal enhances interfacial bonding and facilitates more efficient stress transfer, though the effect remains incremental rather than pronounced. The tanδ curves (Figure 4b) display a relaxation peak associated with the glass transition of the matrix. Peak position and amplitude vary only modestly among the alkali-treated systems, indicating minimal influence of treatment temperature on the bulk mobility of polymer chains. The composites reinforced with fibers treated at 60 °C and 80 °C exhibit marginally lower peak intensities, which is consistent with reduced interfacial friction and the slightly higher E′ values observed in Figure 4a [40]. In contrast, the ABF-100 sample shows a small decrease in modulus together with a modest increase in tanδ amplitude, suggesting that excessive alkaline exposure induces partial damage to the fiber surface and degrades interfacial integrity, leading to a softer overall mechanical response.

### 3.4. Thermal Stability and TGA Analysis

**Figure 5:**
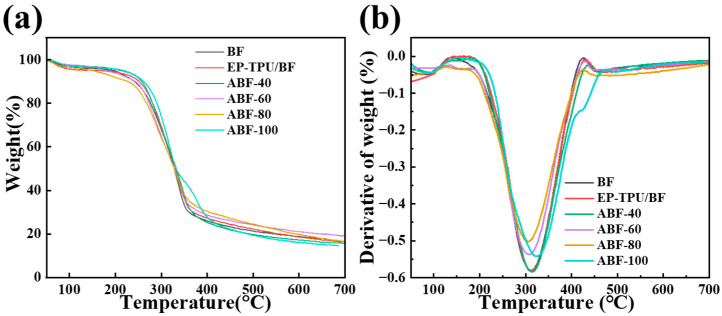
(**a**) TG and (**b**) DTG curves of BF, EP-TPU/BF, ABF-40, ABF-60, ABF-80 and ABF-100.

The thermal degradation behavior was examined under a nitrogen atmosphere., as shown in Figure 5 and Table 2. The BF curve exhibits a major decomposition event between 200 and 350 °C, corresponding to the degradation of hemicellulose and lignin, followed by cellulose depolymerization into levoglucosan (1,6-anhydro-β-D-glucopyranose, a bridged acetal structure) and water [41]. The maximum degradation rate (*T*_max_) occurs at 309.46 °C, and the final char residue is 14.6%. Compared with BF, all EP-TPU/BF and alkali-treated composites show slightly earlier onset of thermal degradation, with decomposition mainly occurring between 150 and 350 °C. The initial decomposition temperatures (*T*_onset_) of ABF-40, ABF-60, ABF-80, and ABF-100 are 243.1, 241.4, 238.5, and 257.6 °C, respectively. Their corresponding *T*_max_ values are 315.48, 310.01, 302.59, and 324.66 °C, and the char residues range from 14.64% to 19.19%. Overall, alkali treatment does not significantly enhance the thermal stability of the composites relative to untreated EP-TPU/BF. A gradual decrease in *T*_max_ is observed with increasing treatment severity up to 80 °C, suggesting that the removal of hemicellulose and lignin results in a more porous fiber structure that is more thermally responsive. The ABF-100 composite shows a slightly higher *T*_onset_ and *T*_max_ than other treated samples, which may be attributed to greater exposure of cellulose hydroxyl groups after extensive alkaline extraction. These hydroxyl groups can participate in additional reactions with epoxy functional groups during curing, contributing to a higher crosslinking density and marginally improved thermal resistance at elevated temperatures.

### 3.5. Hydrophobic Performance of Alkali-Treated Bamboo Composites

**Figure 6:**
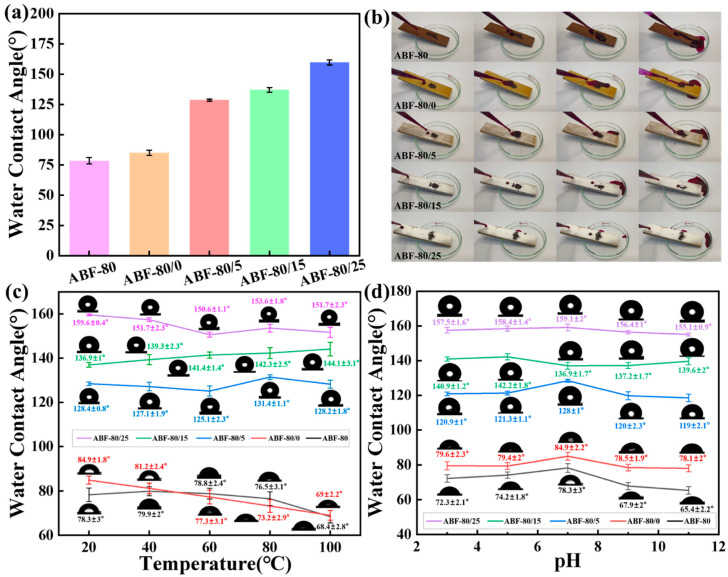
(**a**) Water contact angles, (**b**) Self-cleaning performance, (**c**) Temperature-dependent WCAs and (**d**) WCAs in acidic and alkaline solutions of ABF-80, ABF-80/0, ABF-80/5, ABF-80/15, ABF-80/25.

Figure 6 illustrates the effect of SiO_2_ loading on the wettability, self-cleaning ability, and environmental stability of ABF-80 composites. As shown in Figure 6a, the water contact angle (WCA) increases monotonically with SiO_2_ content. The unmodified ABF-80 exhibits a WCA below 100°, indicative of only mild hydrophobicity. A 5 wt% SiO_2_ coating results in a slight increase but does not generate sufficient surface roughness to significantly alter the wetting state. At 15 wt%, the WCA rises above 130°, corresponding to the formation of a more continuous micro/nanostructured rough surface. The 25 wt% coating achieves superhydrophobicity (WCA > 150°), consistent with a Cassie-Baxter wetting regime in which air pockets trapped between SiO_2_ asperities minimize solid–liquid contact [42]. The combined effect of alkali-induced surface porosity and SiO_2_-generated hierarchical roughness accounts for this substantial enhancement. The self-cleaning behavior (Figure 6b) reflects these wettability changes. ABF-80 and ABF-80/0 allow droplets to spread, leaving most of the methyl red powder adhered. Samples containing 15 wt% SiO_2_ show clear self-cleaning, with rolling droplets removing a majority of surface contaminants. The ABF-80/25 composite exhibits the most effective cleaning, closely resembling the lotus-leaf effect. This performance underscores the correlation between high WCA, reduced solid–liquid contact area, and enhanced particle removal efficiency-key features for outdoor or contamination-prone applications. The thermal robustness of hydrophobicity is shown in Figure 6c. All samples experience a decline in WCA with increasing temperature due to increased droplet spreading and matrix softening. Uncoated ABF-80 and ABF-80/0 exhibit strong deterioration, with WCAs below 70° at 100 °C. In contrast, SiO_2_-coated samples-particularly ABF-80/25-maintain WCAs above 150° across the entire temperature range, demonstrating excellent resistance to thermally induced surface degradation. This behavior can be attributed to the high thermal stability of SiO_2_ nanoparticles and their ability to preserve the hierarchical roughness under thermal expansion. The pH-dependent stability of the modified surfaces is presented in Figure 6d. The uncoated ABF-80 shows substantial WCA losses under both acidic and basic conditions. SiO_2_-coated samples retain much higher hydrophobicity, and the ABF-80/25 composite maintains superhydrophobicity (WCA > 155°) across the entire pH range. The chemical inertness of SiO_2_ and its strong adhesion to the alkali-treated substrate likely contribute to the coating’s resistance to corrosive environments. These results demonstrate that SiO_2_ nanoparticles substantially enhance the hydrophobicity, self-cleaning ability, and environmental resilience of ABF-80 composites, highlighting their potential for outdoor, marine, and chemically demanding applications.

**Figure 7:**
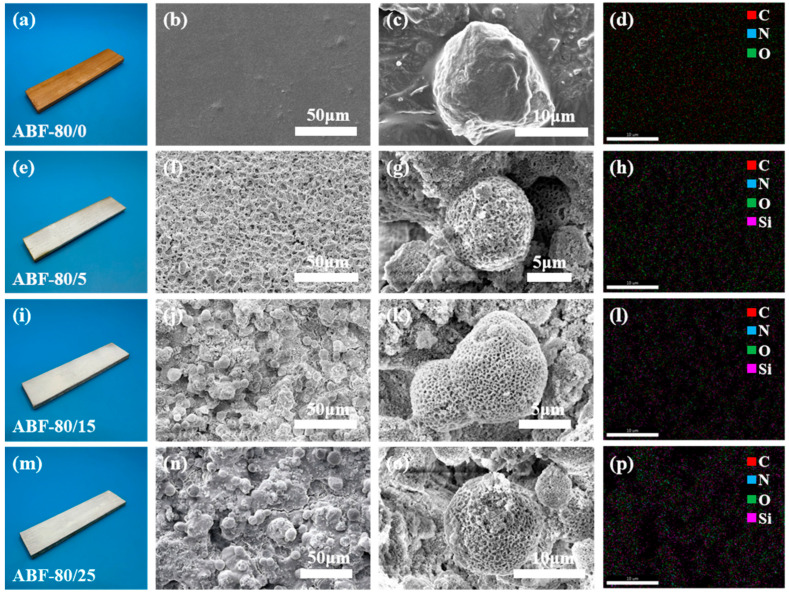
(**a**–**d**) ABF-80/0, (**e**–**h**) ABF-80/5, (**i**–**l**) ABF-80/15, (**m**–**p**) ABF-80/25 Surface photographs, SEM images, and EDS maps of SiO_2_-coated ABF-80 composites.

Figure 7 shows the evolution of surface morphology and elemental composition of ABF-80 substrates coated with increasing amounts of SiO_2_ (0–25%). The unmodified sample (ABF-80/0, Figure 7a–d) presents a relatively smooth surface, and the EDS maps are dominated by C and O signals, consistent with the untreated bamboo–epoxy matrix and its limited hydrophobicity. As the silica concentration increases, both the surface coverage and the distribution density of the particles on the samples exhibit a pronounced increasing trend. At a concentration of 5% (Figure 7e–h), the silica particles are relatively sparsely distributed, and portions of the substrate surface remain clearly visible and Si signals begin to appear across the EDS maps. The coating is continuous but still relatively sparse, which aligns with the moderate improvement in water contact angle observed for this sample. When the concentration is increased to 15% (Figure 7i–l), the particle coverage on the surface becomes more continuous, with a relatively uniform particle size distribution. The Si signal becomes stronger and more continuous, indicating more complete surface coverage. This micro/nanoscale structuring enhances the ability of the surface to trap air and contributes to the higher hydrophobicity of this coating. At a concentration of 25% (Figure 7m–p), the accumulation of surface particles is significantly enhanced, and slight agglomeration can be observed in localized regions, resulting in a denser and more continuous surface-modified layer. The EDS maps show a dense and uninterrupted Si signal, confirming full coverage. Despite some particle agglomeration, the interconnected SiO_2_ network maintains a stable hierarchical texture and limits the formation of capillary pathways. This morphology maximizes air entrapment and lowers the effective solid–liquid contact area, yielding the highest water contact angle among all tested samples. Overall, increasing the SiO_2_ loading promotes the transition from partial to complete coverage and from simple particulate roughness to well-developed hierarchical structures. The ABF-80/25 coating offers the most favorable combination of continuous SiO_2_ domains, multiscale roughness, and reduced liquid–solid contact, resulting in superior hydrophobic performance

## 4. Conclusions

This work demonstrates an effective multiscale modification strategy for enhancing bamboo-based composites by integrating alkali-treated bamboo fibers with a TPU-toughened epoxy matrix and subsequent SiO_2_/FAS-17 surface functionalization. Alkali treatment at 80 °C produced the most favorable fiber structure, improving interfacial bonding and yielding enhanced mechanical performance, with flexural and tensile strengths of 443.97 MPa and 324.14 MPa, respectively. TPU modification contributed to the preservation of structural integrity under elevated temperatures. Surface coatings incorporating SiO_2_ provided tunable wettability, and the ABF-80/25 composite achieved stable superhydrophobicity (WCA > 150°) and reliable self-cleaning behavior. The coatings also demonstrated strong resistance to acidic/alkaline environments and thermal cycling, indicating good long-term durability, demonstrating their suitability for chemically demanding applications such as outdoor structures exposed to acid rain or marine salinity. Overall, the combined fiber treatment, matrix toughening, and surface functionalization approach offers a practical route for developing high-performance and sustainable bamboo composites suitable for structural and outdoor applications. While the bamboo-epoxy substrate is derived from renewable biomass, the superhydrophobic coating utilizes FAS-17. It is acknowledged that fluorinated silanes possess environmental toxicity concerns. However, the significantly extended service life and durability provided by this coating reduce the need for frequent material replacement, thereby contributing to lifecycle sustainability. Future work will investigate fluorine-free alternatives to further improve environmental compatibility.

## Figures and Tables

**Table 1:** Mechanical properties of BF, EP-TPU/BF, ABF-40, ABF-60, ABF-80, and ABF-100.

Materials	Tensile Strength (MPa)	Bending Strength (MPa)	Elastic Modulus (GPa)
BF	93.97 ± 2.4	154.57 ± 10.15	7.9 ± 0.31
EP-TPU/BF	198.09 ± 5.11	208.2 ± 18.21	9.7 ± 1.53
ABF-40	185.58 ± 10.35	212.35 ± 24.90	10.2 ± 1.79
ABF-60	273.57 ± 16.96	394.95 ± 29.43	11.6 ± 2.52
ABF-80	324.14 ± 20.12	443.97 ± 22.16	14.4 ± 2.34
ABF-100	135.24 ± 35.87	235.12 ± 30.91	9.1 ± 2.71

**Table 2:** Tg of BF, EP-TPU/BF, ABF-40, ABF-60, ABF-80, and ABF-100.

Materials	*T*_ONSET_ (°C)	*T*_MAX_ (°C)	Residual Rate (%)
BF	243.5	309.4	14.6%
EP-TPU/BF	251.1	312.0	16.6%
ABF-40	243.1	315.4	15.8%
ABF-60	241.4	310.0	19.2%
ABF-80	238.5	302.5	16.7%
ABF-100	257.6	324.6	14.6%

## Data Availability

The data that support the findings of this study are available from the corresponding author upon reasonable request.

## Supplementary Materials

The following supporting information can be downloaded at: https://www.mdpi.com/article/10.3390/nano16010008/s1, Table S1. Experimental materials and chemicals.
