# Supplementary material for: Multifunctional Bamboo Fiber/Epoxy Composites Featuring Integrated Superhydrophobicity and Enhanced Mechanical–Thermal Performance
Source: Nanomaterials (Basel). 2025 Dec 19;16(1):8. doi: 10.3390/nano16010008 (PMC12787842; doi:10.3390/nano16010008)
Supplement: Supplementary file 1 [file nanomaterials-16-00008-s001.zip › nanomaterials-4047784-supplementary.pdf]

Supporting Information

Multifunctional Bamboo Fiber/Epoxy Composites Featuring Integrated Superhydrophobicity and Enhanced Mechanical-Thermal Performance

Yanchao Liu [1, a], Ze Yu [1, a], Rumin Li [1, *], Xiaodong Wang [1, *], Yingjie Qiao [1] and *

[1] College of Material Science and Chemical Engineering, Harbin Engineering University, Harbin 150001, China. Yanchao Liu and Ze Yu, contributed equally to this work and should be considered as co-first authors. liuyanchao2024@163.com；yuze1996@hrbeu.edu.cn

* Correspondence: ruminli2024@163.com; wangxiaodong@hrbeu.edu.cn; qiaoyingjie2024@163.com

Experimental Materials and Methods

The raw materials and chemicals used are listed in Table 1. All chemicals were used directly as received.

Table S1. Experimental materials and chemicals.

Experimental materials	Specifications	Supplier
Moso bamboo	-	
E-51 Epoxy Resin (EP)	A.R.	Suzhou Qicai Stone Composite Materials Co., Ltd.
4, 4'-Diaminodiphenylmethane (DDM)	A.R.	Shanghai McLean Biochemical Technology Co., Ltd
Acetone	A.R.	Hefei Kaimike Biochemical Technology Co., Ltd.
Polyurethane (TPU-1185A)	A.R.	Badische Anilin-und-Soda-Fabrik
N, N-dimethyllformamide (DMF)	A.R.	Shanghai McLean Biochemical Technology Co., Ltd
Epichlorohydrin	A.R.	Badische Anilin-und-Soda-Fabrik
Sodium hydroxide (NaOH)	A.R.	Shanghai McLean Biochemical Technology Co., Ltd
Tetramethylammonium bromide	A.R.	Shanghai McLean Biochemical Technology Co., Ltd
Ethyl acetate (EA)	A.R.	Tianjin Fuyu Fine Chemical Co., Ltd
1H,1H,2H,2H-Heptadecafluorodecyltrimethoxysilane (FAS-17)	A.R.	Shanghai McLean Biochemical Technology Co., Ltd
Fumed silica		Shanghai McLean Biochemical Technology Co., Ltd

Preparation of Alkali-Treated Bamboo （ABF）

A total of 10 g of bamboo was immersed in 250 mL of 0.2 mol/L sodium hydroxide (NaOH) solution for 12 h. After treatment, the bamboo was thoroughly rinsed with deionized water until the washings became neutral, and subsequently dried in a vacuum oven at 40 °C for 12 h to remove residual moisture.

For epoxidation, 10 g of the alkali-treated bamboo was transferred into a reaction vessel containing 171.53 g of epichlorohydrin and 13.35 g of solid NaOH. The mixture was stirred at 200 rpm under a nitrogen atmosphere for 30 min. Then, 0.5140 g of tetramethylammonium bromide was added, and the reaction was maintained at 65 °C for 6 h

under constant stirring. Upon completion, the bamboo was retrieved and washed three times with absolute ethanol and three times with distilled water. Finally, the bamboo was dried in a freeze-dryer for 12 h to obtain epoxidized bamboo.

Preparation of TPU-Toughened Epoxy Resin

Predetermined amounts of EP, TPU, DDM, and DMF were weighed. EP was preheated at 80 °C for 10 min in an oven to reduce viscosity. TPU was first dissolved in DMF with continuous stirring, followed by the addition of EP. After the mixture became homogeneous, DDM was introduced as the curing agent and stirred until uniform.

The solution was then subjected to vacuum degassing for 30 min to eliminate entrapped air bubbles. After degassing, the mixture and custom mold were weighed on an analytical balance to monitor solvent removal during evaporation. The mold was preheated and leveled with a spirit level to ensure uniform distribution of the resin during solvent evaporation. The homogeneous solution was poured into the preheated mold and allowed to evaporate at 60 °C. The samples were weighed every 2 h until the solvent was completely removed. The solvent-free resin was subsequently sealed and stored for further use.

Preparation of EP–TPU/BF Composites

BF was impregnated with EP–TPU under vacuum at 40 °C for 90 min. The treated BF was then placed into a mold with a target thickness of 2.5 mm. Hot-press molding was carried out at 120 °C and 8 MPa for 5 h to remove the solvent and promote curing. After hot pressing, the composites were allowed to stand for 12 h, demolded, and obtained as EP–TPU/BF composites.

Preparation of Hydrophobic Coating on Composites

A total of 0.5 g FAS-17 was added into 50 g EA and stirred for 2 min. Subsequently, 4 g EP and 1 g DDM were introduced into the above solution and stirred for an additional 5 min. After homogenization, different mass fractions of fumed silica (0%, 5%, 15%, and 25%) were added, followed by ultrasonic treatment for 20 min. The resulting suspension was uniformly sprayed onto the surface of the pre-fabricated composite specimens using a spray gun and then dried in an oven at 80 °C for 8 h, yielding composites with a hydrophobic surface layer.

Characterization

(1) The chemical properties of the samples were characterized using a Fourier Transform Infrared (FT-IR) spectrometer (Tensor II, Bruker, Hong Kong). The spectra were collected over a wavenumber range of 4000 to 500 cm^{-1}, with a scanning frequency of 32 scans.
(2) X-ray photoelectron spectroscopy (XPS, K-Alpha, Thermo Fisher Scientific Co., Ltd., USA) was employed to characterize the elemental composition of the composites. The excitation source utilized was Al Kα, with a spectral energy range from 1350 eV to 0 eV and a scan increment of 1 eV. During the testing process, samples were collected from the center of the mechanical fracture surfaces.
(3) Mechanical performance

Three-point bending test: A universal mechanical testing machine (CMT-5504, SANS Testing Machine, Shenzhen, China) was utilized to assess the three-point bending strength of the samples. A minimum of six specimens were tested for each composite material. The dimensions of the bending test specimens were 100 × 20 × 2.5 mm^3, and the tests were conducted at a loading speed of 1 mm/min.

Tensile performance test: The tensile properties of the composites were tested according to the method specified in GB/T 2567-2008, using a universal testing machine (model UTM4304, Shenzhen SANS Testing Co., Ltd.). The specimen dimensions used in this experiment were 10 × 100 × 2.5 mm³, and the tests were conducted at a tensile speed of 1 mm/min.

The formula for calculating tensile strength is as follows:

$$\sigma_t = \frac{P}{b \times h} \tag{S1}$$

σ_t—— Tensile strength of resin samples (unit: MPa);
P——The maximum load that the resin cast sample can withstand (unit: N);
b——The width of the resin cast sample (unit: mm);
h——The thickness of the resin casting experiment (unit: mm)。

The formula for calculating the elongation at break:

$$\gamma_1 + cos\,\theta = 2\sqrt{\gamma_1^p \gamma_s^p} + 2\sqrt{\gamma_1^d \gamma_s^d} \tag{S2}$$

$$\varepsilon_1 = \frac{L - L_0}{L_0} \tag{S3}$$

ε_t—— The elongation at break of the resin casting sample (unit: %);
L——The fracture gauge length of the resin casting sample when it breaks (unit: mm);
L_0—— Original gauge length of resin cast specimen (mm)。

For each adhesive mixture system, five parallel tests were conducted on the resin-cast specimens. The average of the data from these five tests was considered the final result.

(4) Morphology observation: A field emission scanning electron microscope (SEM, Quanta 220, FEI, USA) were operated to record the microscopic structure of samples.

(5) The crystalline structure of the composites was characterized using an X-ray diffractometer (Rigaku Corporation, Japan). Measurements were carried out at a scan rate of 5 °/min, with an operating voltage of 30 kV and a current of 20 mA. The diffraction angle (2θ) was scanned from 5° to 60° with a step size of 0.02°. Composite specimens with dimensions of 20 × 20 × 2.5 mm³ (length × width × thickness) were used for testing.

(6) Water contact angle (WCA): The WCA of the samples was measured using a contact angle goniometer (OCA20), with a minimum of six measurements taken for each sample.

(7) Thermal stability: Thermal stability of samples was conducted using a thermogravimetric analyzer (TG, NETZSCH, Germany) by gradually increasing the temperature from 30 °C to 700 °C at a rate of 10 °C/min.

(8) Differential scanning calorimetry (DSC) testing: DSC curve was performed using a differential scanning calorimeter (DSC, DSC-500, USA). A sample of 5-10 mg was placed in an aluminum crucible and heated from 25 °C to 300 °C at a heating rate of 10 °C/min under a nitrogen atmosphere (50 mL/min).

(9) The viscoelastic behavior of the composites was characterized using a dynamic mechanical analyzer (DMA 242 E, Netzsch, Germany) in a three-point bending mode. All samples were scanned over a temperature range of 25 to 200 °C with a controlled heating rate of 5 °C/min and a constant frequency of 1.0 Hz. Prior to testing, samples measuring 50 mm × 10 mm × 2.5 mm (length × width × thickness) were dried overnight.

(10) Methyl red powder was used to simulate environmental dust and was uniformly spread on the sample surface; subsequently, a droplet of alkaline violet solution was dropped onto the surface to evaluate the self-cleaning performance.

(11) To examine the thermal resistance, samples were immersed in water at 0, 20, 40, 60, 80, and 100 °C for 2 h, then dried in air, and the contact angle was measured.

(12) The acid–base resistance was assessed by immersing the samples in acidic (CH_3COOH, pH = 3 and 5), neutral (NaCl, pH = 7), and alkaline (NaOH, pH = 9, 11, and 13) solutions for 2 h. After immersion, the samples were dried in an oven at 80 °C for 2 h, and contact angles were measured at three different positions.
